# Antipyrine

**Published:** 1887-05

**Authors:** 


### ANTIPYRINE.

A subscriber asks to be informed in relation to the history and literature of antipyrine. As yet, but little has been published in regard to the mode of its preparation and its history. Technically, it is dimethyl oxychinozin. It is a quinoline compound, and was discovered by Dr. Knoor, of Erlangen, Germany. Dr. Filehne, a German writer, was the first to publish a full account of it. As we have not his work before us, we cannot give particulars as to the method of its manufacture, and how it was first discovered. Its exact name is not yet determined. It is a reddish-gray powder, very soluble in water, and has a slightly bitter taste. It has been given by the mouth in doses of 10 to 30 grains, and may be given hypodermically or by enema. In overdoses it acts somewhat like styrchnia, causing tetanic symptoms. It is supposed to reduce the temperature by acting upon the thermogentic or heat centres. We would be pleased to have reports from medical brethren as to the use and properties of this new and valuable agent.

---

WE are pleased to note the publication of the contribution of Dr. J. McF. Gaston to the British Medical Association, at Brighton, on the practicability of accomplishing a fistulous communication in the human subject from the gall-bladder into the duodenum. This well illustrated paper appeared in the British Medical Journal of February 5, and we learn that Professor Gaston has a letter of indorsement from Dr. George Hosley, of London—the highest authority upon disorders of the liver in the medical world. Mr. Willet also presented a paper before the British Medical Association, advocating the feasibility of this cholecysto-duodenal opening, while Golzi, an Italian surgeon, has reported a series of experiments upon dogs, corroborating those made by our Atlanta surgeon for demonstrating this outlet for the bile in cases of complete impermeability of the gall duct. Thus, it would seem, that the claim of Dr. Gaillard for this mode of communication between the gall-bladder and duodenum as "Gaston's Operation" is sustained by prominent members of the medical profession abroad, and that this measure of relief is likely to be adopted in cases of entire occlusion of the common bile duct.

